# Innovative use of a syringe needle cover in hair transplantation

**DOI:** 10.1111/srt.13747

**Published:** 2024-05-08

**Authors:** Tao Wang, Yeqin Dai

**Affiliations:** ^1^ Department of Dermatology Hangzhou Third People's Hospital,Hangzhou Third hospital affiliated to zhejiang chinese medical university Hangzhou Zhejiang China

When conducting hair transplantation procedures, various tools, including gem knives, syringe needles, and implanting pens, are commonly employed. Gem knives, although sharp, typically result in relatively lower levels of implantation density. In contrast, implanting pens and syringe needles allow for higher implantation density. Accurately controlling implantation depth remains challenging when using gem knives, syringe needles, and implanting pens, primarily relying on the skill of the operating surgeon.

## SOLUTION

**FIGURE 1:**
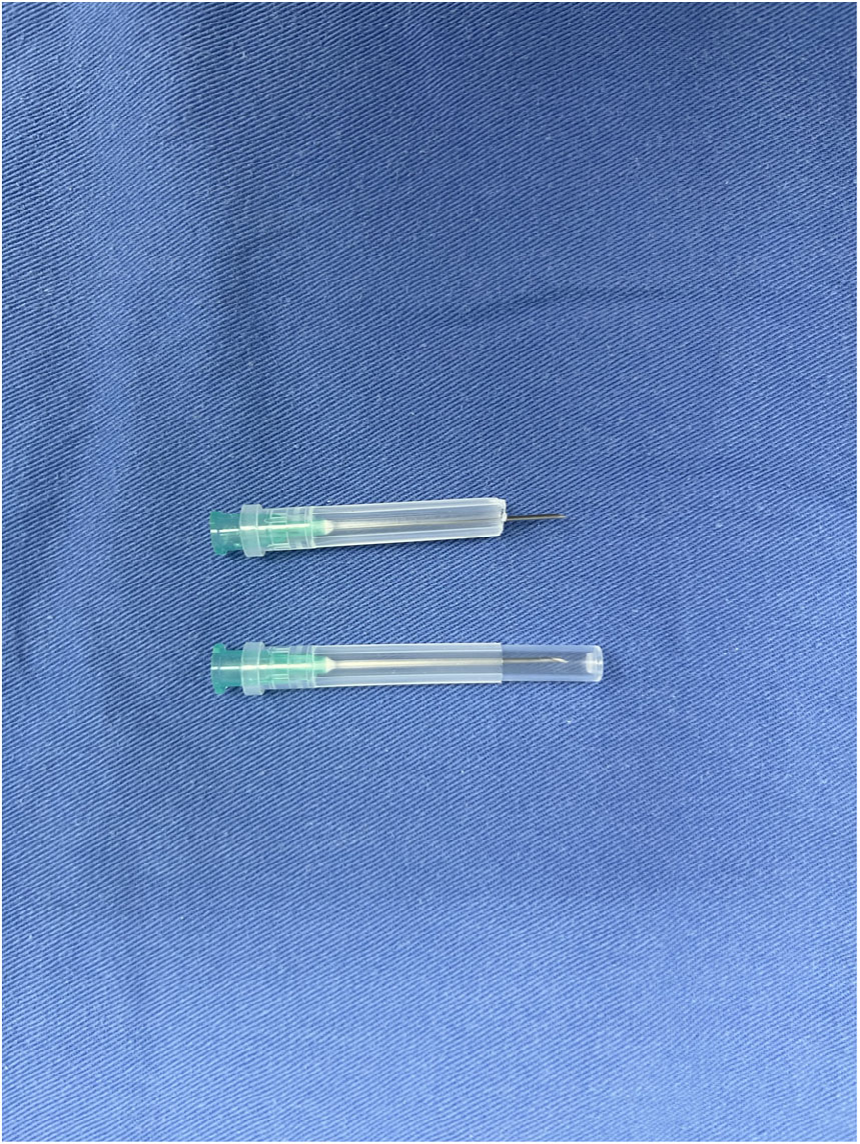
Syringe needles and their protective sleeves for preparation.

**FIGURE 2:**
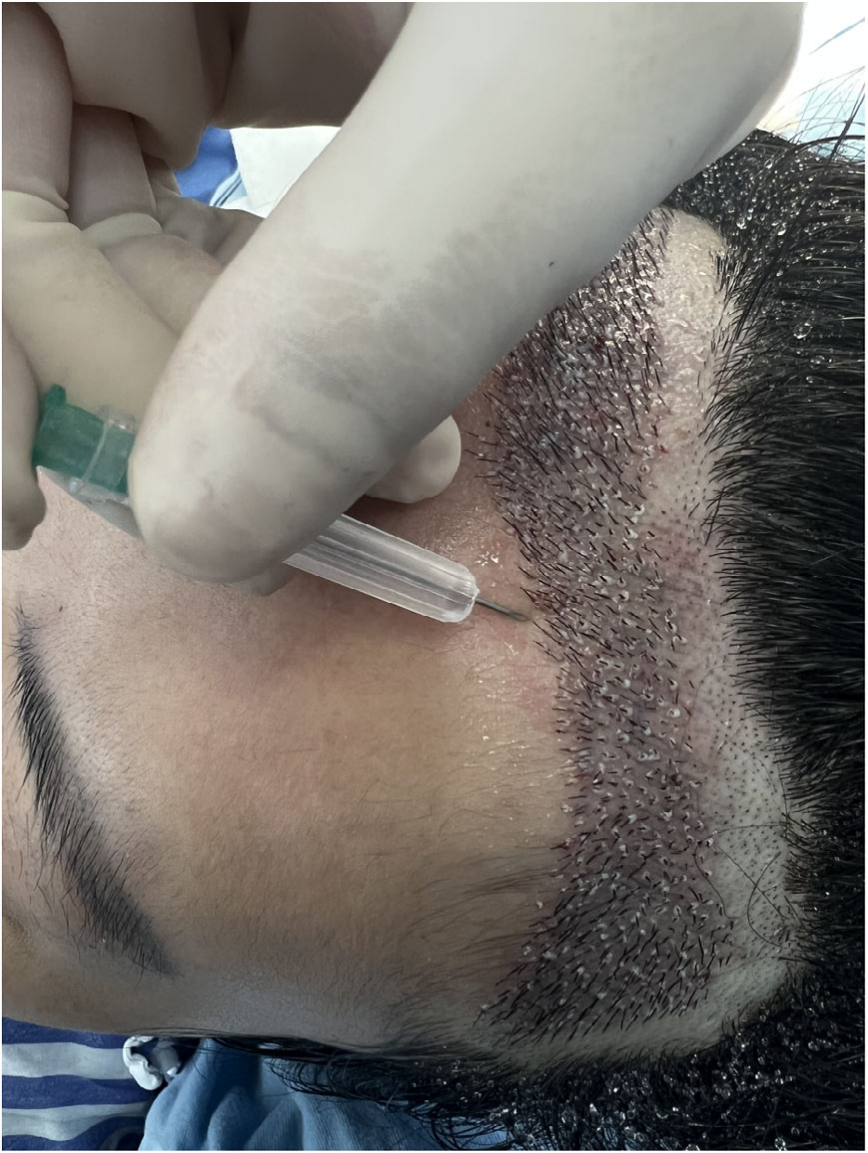
Hand‐held syringe needles for puncturing and hair transplantation.

We discovered that utilizing the syringe needle cover effectively addressed the challenge of controlling implant depth during hair transplantation. A suitable syringe model can be chosen depending on the number of hairs in the hair follicle unit. For implanting a single hair, an 18G needle can be used, while a 16G needle can be employed for the implantation of two to three hairs. The end of the needle cover can be trimmed according to the depth of the implanted hair follicle, ensuring a consistent implantation depth that is not reliant on the subjective judgments of the operating surgeon (Figure 1). The needle cover can also provide protection for the surgeon, minimizing the risk of puncture wounds, while simultaneously enhancing hand comfort during the procedure (Figure 2).

## CONFLICT OF INTEREST STATEMENT

The author reports no conflicts of interest. The author is responsible for the content and writing of the paper.

## PATIENT CONSENT STATEMENT

Consent for the publication of recognizable patient photographs or other identifiable material was obtained by the authors and included at the time of article submission to the journal stating that all patients gave consent with the understanding that this information may be publicly available.

## Data Availability

The data that support the findings of this study are available from the corresponding author upon reasonable request.

